# A literature review of knowledge translation and partnership research training programs for health researchers

**DOI:** 10.1186/s12961-019-0497-z

**Published:** 2019-12-16

**Authors:** Hannah Tait, Anna Williamson

**Affiliations:** The Sax Institute, PO Box K617, Haymarket, NSW 1240 Australia

**Keywords:** Knowledge translation, partnership research, training

## Abstract

**Background:**

Researchers and policy-makers are increasingly working together with the goal of creating research that is focused on solving real-world problems; however, knowledge translation (KT) activities, and the partnerships they often require, can be challenging. The aim of this review is to determine the extent of the literature on training programs designed to improve researcher competency in KT and to describe existing training methods that may be used by those hoping to build capacity for partnership research.

**Methods:**

MEDLINE, EMBASE, PsycINFO and CINAHL were searched for peer review articles published between January 2000 and July 2019. Studies were eligible for inclusion in the review if they described the development of, curriculum for, or evaluation of KT and/or partnership research training programs. Data extraction included information on evaluation methods, outcomes and implications as well as the format, aims and themes of each capacity-building program.

**Results:**

The review identified nine published articles that met inclusion criteria – four papers described training events, two papers described participant experiences of specific learning sessions within a larger training course, two papers described part time secondments for KT capacity-building and one paper described a plan for KT training embedded within an existing research training course. All programs were delivered face-to-face, all included practical skills-building opportunities, and all employed multiple learning modalities such as seminars and small group discussions. Evaluation of the training programs was primarily conducted through qualitative interviews or feedback surveys.

**Conclusion:**

To date, few KT training initiatives have been described in the literature and none of these have been rigorously evaluated. The present review offers insights into the planning, development and participant experiences associated with the small number of training initiatives that have been described. There is insufficient evidence available at present to identify the most effective models for training researchers in KT and partnership skills.

## Background

Funding bodies and researchers internationally are increasingly interested in ensuring that research has real-world impact, informing policy and/or practice [1–4]. The field of knowledge translation (KT) is concerned with how to bridge the research/research-user divide [5] and has been defined by the Canadian Institutes of Health Research as the “*exchange, synthesis and ethically-sound application of knowledge –within a complex system of interactions among researchers and users – to accelerate the capture of the benefits of research*” [2]. Common KT activities undertaken by researchers include disseminating research findings to policy-makers and creating evidence summary materials that are accessible and relevant to a policy audience [6, 7]. Research has also been undertaken to determine how best to build the capacity of policy-makers to engage with research and to understand the barriers they face in doing so [8–10].

More recently, those advocating for reductions in research wastage [11] have asserted that communicating the end products of research clearly for a policy audience is not enough to enhance translation if the evidence in and of itself is not relevant to the interests and needs of policy-makers and practitioners. Partnering with potential research users throughout the research process has been posited as a potentially effective method of improving the policy and practice relevance of research [12, 13]. This style of research, where there is ongoing engagement between decision-makers (who bring with them contextual and tacit knowledge about what evidence is needed in practice) and researchers (who bring skills in research methodology), is becoming increasingly popular. Such collaborations are thought to increase the likelihood of evidence translation because policy-makers are involved in determining the research question (increasing relevance) and in the evidence-generation process (increasing the extent to which they are aware of and understand the findings) [13–17]. This mode of research, which places a strong emphasis on partnership, is often termed integrated KT (IKT) [14, 15, 18].

Improving the use of evidence in policy through increased collaboration with researchers is a deceptively simple idea; however, partnership research activities are often met with challenges resulting from the different needs, expectations and cultures in each professional context [3, 19–21]. In recognition of these challenges, interest in building capacity for effective KT and IKT work has been steadily growing [22]. A key contribution in this area was Straus et al.’s [22] mapping of four core competencies in KT, namely understanding models and theories of KT and KT research, capacity to conduct synthesis, capacity in KT research methods, and ability to evaluate the impact, effectiveness and sustainability of KT strategies. More recently, Mallidou et al. [23] built on these competencies and, after completing a scoping review that included grey literature and KT job descriptions, identified 19 competencies. They grouped these competencies into three categories as ‘knowledge’, ‘skills’ and ‘attitudes’. Some themes identified under ‘knowledge’ and ‘skills’ included understanding the context, understanding the research process, understanding translation and dissemination activities, dissemination of research findings, knowledge brokering, fostering innovation, and use of research findings.

Understanding the competencies required to succeed in KT work provides a framework to understand KT training activities. The focus of this paper is published literature about training courses designed to build capacity in KT, IKT or partnership research that involved health researchers.

The aims of this study are two-fold. Firstly, to describe any KT and/or IKT partnership training directed at health researchers with respect to the methods of delivery, course content and training themes. Secondly, to examine the evaluation approaches used to establish the effectiveness of the training in achieving KT knowledge and skills.

## Methods

### Search strategy

**Fig. 1:**
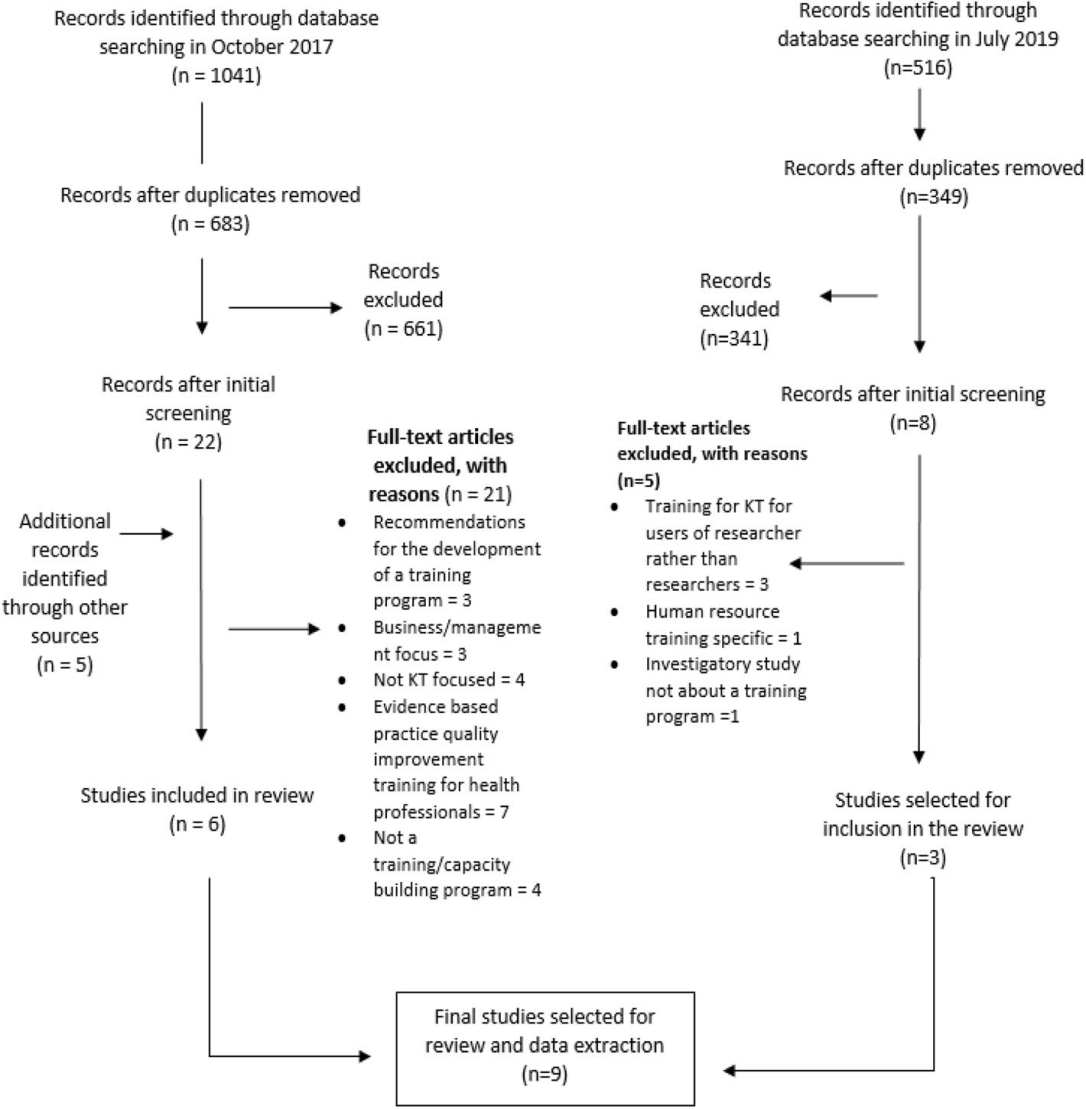
Prisma diagram of eligible studies. Flow diagram of included and excluded studies from the database search of MEDLINE, EMBASE, PsycINFO and CINAHL, with reasons for exclusion

A broad initial literature search was performed to establish major themes and to develop the final search strategy. MEDLINE, EMBASE, PsycINFO and CINAHL were searched for peer review articles published between 2000 and 2017 in October 2017 and articles published between 2017 and 2019 on 1 July 2019. A Boolean search of abstracts was performed using the terms ‘knowledge translation’ OR ‘integrated knowledge translation’ OR ‘knowledge transfer’ OR ‘co-produc* research’ OR ‘partner* research’ AND training using truncation (indicated by *) to allow for multiple word endings (e.g. co-produced research or co-production). The results were limited to humans and articles published in English between 2000 and 2019. The initial search returned 1041 and 516 results, respectively. After duplicates were removed in Endnote, there were 683 titles and abstracts to review in 2017 and 349 titles and abstracts to review in 2019 (Fig. 1).

### Process for study selection

Initially, 30 papers were identified as potentially relevant (22 in 2017 and a further 8 in 2019), and reference lists of selected studies were scanned to identify relevant articles that may not have been captured in the initial search. Studies included in the review were peer reviewed articles describing the development, curriculum for, or evaluation of KT and partnership research training programs (using the most inclusive definition of these terms possible, as per the search terms listed above). The inclusion criteria were kept broad so as to include as many papers as possible, which might have relevant information pertaining to training of researchers in KT. Studies were excluded if they described a KT intervention or a training course focused on improving evidence-based practice among health professionals. Studies assessing KT training needs (to inform future training programs) or mentorship programs were beyond the scope of this review [24–26]. Studies describing training programs for dissemination and implementation science [27, 28] were also excluded.

### Data extraction

In order to describe the training initiatives about which published literature was available, we extracted information pertaining to the country in which the training was conducted, training participant type (e.g. researchers, policy-makers), the duration and modality of the training activity, and the aims and focus areas addressed by each initiative. In addition, we generated a brief description of each training activity.

In order to assess both the outcomes of the reported evaluations and their quality, we extracted information on the number of participants, the evaluation methods used, the outcomes of the evaluation and the reported implications of the evaluation, and assessed what level of evidence each evaluation reported according to National Health and Medical Research Council (NHMRC) level of evidence standards [29]. The Australian NHMRC’s level of evidence hierarchy is commonly used in systematic reviews and clinical guidelines. It consists of four levels of evidence (I, II, III-1, III-2, III-3 and IV) with evidence from a systematic review of randomised controlled studies having a rating of I and cohort studies having a rating of III-2.

## Results

The review identified nine published reports describing KT capacity-building activities for researchers. The studies came from Canada (*n* = 3), Nigeria (*n* = 3), America (*n* = 1), Australia (*n* = 1) and England (*n* = 1). The aims and training approaches of each program are outlined in Table 1. Three papers describe different aspects of the annual Knowledge Translation Summer Institute held in Canada [36–38], Kho et al. [36] outline the development and curriculum of the 2008 program, and two other papers provide informal descriptions of single training sessions presented in 2009 [37, 38].

**Table 1:** Capacity-building initiatives described by the papers included in the review

	Authors, year, reference	Country	Participants	Duration	Capacity-building modality	Aims of initiative/key areas covered/description of training program
	Gerrish & Piercy (2014) [30]	United Kingdom	Clinical and academic nurses from pilot organisations who undertook secondments from 2009 to 2012	9–24 months part time (0.2 FTE)	Secondments into KT project teams	Aims not specified Secondments were created to enhance the expertise in KT teams, especially clinical and evaluation skills (from the academic secondees) and provide capacity-building opportunities to benefit CLAHRC partners in the long term Key skills included applying KT frameworks in practise, evidence appraisal, skills to facilitate practice change and evaluation
	Jones et al. (2015) [31]	Australia	Researchers	1 day	Training course (face-to-face)	Key themes: (1) KT and exchange theory and science; (2) planning for KT; (3) developing relationships for engagement and exchange with decision-makers; (4) communicating research; and (5) evaluating KT and research impact
	Santacroce et al. (2018) [32]	United States of America	University of North Carolina pre- and postdoctoral research nurses	Training and competency assessment activities were embedded into pre- and post-doctoral coursework and research outputs over several years	Training is integrated into coursework, mandatory scientific seminars and other research training activities	Key themes include stakeholder engagement, patient-centred outcomes, intervention optimisation and sequential multiple randomized trials (SMART), pragmatic trials, mixed methods approaches, and dissemination and implementation science strategies Students are required to demonstrate proficiency in two of the key research translation themes in their scholarly products
	Uneke et al. (2012) [33]	Nigeria	Policy-makers, researchers and other major stakeholders	1 day	One-day workshop	The workshop was designed to facilitate the meeting of health researchers and policy-makers in Nigeria, and to assist those working on similar projects to overcome professional barriers between the two disciplines. Lectures and interactive discussions were held around the following topics: 1) Introduction to health policy and systems research/building evidence-informed policy environments; (2) Capacity development and enhancement for evidence-informed health policy-making; (3) Acquisition, assessment, adaptation & application of evidence in health policy-making and (4) Building effective linkage, partnership & exchange between health policy-makers and researchers in Nigeria. Participants in the workshop were also broken up into six focus groups to discuss the topic ‘Bridging the gap between health policy-makers & researchers’
	Uneke et al. (2018) [34]	Nigeria	10 researchers and 10 senior policy staff	Part-time secondment, up to 2 working days per week over a period of 6 months	Two-way secondment (policy-makers working in research teams and researchers working within government health programs)	Researchers seconded to policy teams provided research expertise to several projects, including the State Malaria Elimination program, reproductive health services, and primary healthcare Research secondees were instructed to (1) build trust and understand policy-maker’s evidence needs; (2) play an expert advisory role and provide scientific evidence to guide on policy issues; and (3) provide capacity enhancement for policy-makers Policy-maker experiences (in research organisations) were also reported; however, this is out of scope for the present review Ultimately, the two-way secondment aimed to increase collaboration between policy-makers and researchers in Nigeria and build capacity for ongoing evidence-informed policy-making Following the secondments, all participants (both researchers and policy-makers) attended a policy dialogue event where they received training on preparing a policy brief; the event was used to produce national guidelines on malaria control using insecticide-treated nets in Ebonyi State
	Uneke et al. (2018) [35]	Nigeria	45 participants: researchers from the Implementation Research Team, policy-makers (from the Ministry of Health, Local Government Area, state primary healthcare development agency) and representatives from non-governmental organisations	3 days	Training course (face-to-face)	The aim of the training program was to increase the capacity of policy-makers and researchers to undertake KT and promote evidence-based policy; the 3-day training workshop included 15 modules (5 per day) The 15 modules were Introduction to health policy and health systems; Introduction to KT (IKT and End-of-Grant KT); Research priority setting; Leadership capacity development and managing political interference; Getting research into policy and practice; KT models measures; Research evidence in health policy-making and health policy implementation; Health policy advocacy, demand creation, consensus-building and negotiations; KT tools and strategies for stakeholders and end users engagement; Policy formulation and implementation process. Modules were taught each day by way of lectures and group work sessions; lecture sessions used learning tools such as PowerPoint slides and handouts; group work consisted of focus group discussion, question/answer sessions and group work
Papers emerging from the KTSI run by the Canadian Institutes of Health Research	Kho et al. (2009) [36]	Canada	Primarily doctoral students or PhDs (early career researchers)	4 days	Training course (face-to-face)	The aims of the KTSI in relation to health services, policy, population or public health were (1) to explore the challenges of planning and carrying out KT research and KT; (2) to explore involving and/or engaging different stakeholder groups; (3) to increase the understanding of concepts, methods and theories relevant to KT research; and (4) to investigate the contribution of different disciplinary and methodological approaches to KT practise
	Leung et al. (2010) [37]	Canada	One group of participants from the 2009 KTSI (doctoral students and early career researchers)	One learning session	Component of training course (practice developing an end-of-grant KT plan)	The aims of this session were to provide participants with an opportunity to develop an end-of-grant KT plan for multiple stakeholders and have exposure to the challenges of the KT planning process in a supervised environment; in developing the plan, the participants used a three-step process to inform the key strategies for the project, identifying first the goals of the KT plan, and from this the target audience and key messages; participants also planned out dissemination and diffusion activities
	Bhogal et al. (2011) [38]	Canada	One group of participants from the 2009 KTSI (doctoral students and early career researchers)	One learning session	Component of training course (problem-based learning case study – developing a KT intervention)	This problem-based learning session contributed to the goals of the KTSI by providing opportunities for trainees to learn the skills necessary to carry out a KT intervention and to give students exposure to the challenges, variety and complexity of KT cases

*CLAHRCs* Collaborations for Leadership in Applied Health Research and Care; *FTE* full time equivalent; *IKT* integrated knowledge translation; *KT* knowledge translation; *KTSI* Knowledge Translation Summer Institute

### Participants, duration and training modality

The identified capacity-building initiatives all included researchers as participants; however, a range of other professionals were also included. Gerrish and Piercy’s [30] secondment program included clinical nurses, while Uneke et al. [33–35] included policy-makers, healthcare managers, directors of NGOs and other stakeholders alongside researchers in their initiatives. Participants in the Canadian Summer Institute, from which three papers emerged [36–38], were all researchers or graduate students interested in KT research. The duration of the training initiatives varied from part time secondments, which lasted between 6 months [34] and up to 2 years [30], to Santacroce et al.’s [32] program, which was embedded into existing training programs for nurse scientists over several years (pre- and post-doctoral coursework). The other programs [31, 33, 35, 36] were brief (between 1 and 4 days) face-to-face courses or were delivered during a single session [37, 38].

### Aims of the training programs

All programs aimed to build the capacity of trainees in KT; however, how this was operationalised differed. The program reported by Gerrish and Piercy [30] aimed to build skills in applying KT frameworks in practise, evidence appraisal, skills to facilitate practice change and evaluation. Jones et al. [31], Kho et al. [36] and Uneke et al. [35] all focused on theory of KT and planning for KT. Santacroce et al. [32] proposed a framework of six research translation themes (dissemination oriented) embedded into an existing research curriculum. The workshop [33] and secondment program [34] described by Uneke et al. aimed to facilitate networking between researchers and policy-makers and to build capacity for evidenced-based policy work. The session reports by Leung et al. [37] and Bhogal et al. [38] described the aim as being to increase participant skills and confidence in developing KT strategies.

### Training program activities/curricula (practical skills-building, relationship-building and theoretical knowledge)

All the training initiatives identified included skills-building elements in their programs. The most common focus of skills development was KT intervention planning, included in Jones et al. [31], Kho et al. [36], Gerrish and Piercy [30], Uneke et al. [35], and Santacroce et al. [32].

Relationship building with policy-makers was another common theme, addressed in five of the nine programs [30–34]. For example, Jones et al. [31] explored “*developing relationships for engagement and exchange with decision-makers*” in lecture form. In the most recent program described by Uneke et al. [34], a society for “*health policy research and knowledge translation*” was established to facilitate ongoing collaboration between researchers and policy-makers. All training programs offered the opportunity for professional networking during the training event.

Two additional foci that emerged as capacity-building strategies in this review were providing information about the theoretical background of KT and specific training in KT research. Specific sessions on KT theory were delivered in three out of nine initiatives [31, 35, 36]. The Knowledge Translation Summer Institute described by Kho et al. [36] had a strong focus on developing researchers’ skills in KT research methods and theory, with sessions such as ‘Evaluating knowledge translation interventions’ and ‘Ethics of knowledge translation and knowledge translation research’. This 4-day course also offered participants mentoring and practical small group exercises to assist their learning. In these sessions, practise-based learning exercises were used to assist students in thinking through the challenges of developing an end-of-grant KT plan [37] or a KT intervention for a clinical scenario [38] while having supervision, peer support and access to relevant resources.

### Evaluation methods and measures

Table 2 outlines the evaluation approaches, outcomes and implications reported in each of the papers that met selection criteria. NHMRC grade of evidence is also reported alongside this synthesis of findings. The programs from the United Kingdom [30], Australia [31] and Nigeria [33–35] all reported varying degrees of formal evaluation. The paper from the United States reports planned evaluation measures [32]. The reports coming out of Canada [36–38] do not report evaluation of the programs but do discuss participant experiences.

**Table 2:** Evaluation details from reports included in the review

Authors, year, reference	Participants	Evaluation method	Outcomes	Implications	NHMRC Level of Evidence
Gerrish & Piercy (2014) [30]	Participants in the evaluation came from three groups: secondees (10 nurses (5 clinical and 5 academic) and 4 dieticians), seconding organisations and host CLAHRC teams	Qualitative, post-secondment self-report on impacts Phase 1: Focus groups with secondees: academic (*n* = 5), clinical (*n* = 5) Semi-structured interviews with the 4 remaining secondees and managers from healthcare (*n* = 2), university (*n* = 2) and KT projects (*n* = 3) Individual and focus group interview schedules covered participants’ views regarding the ‘success’ of secondments and how this was judged, reasons for supporting or undertaking secondments, and factors influencing success of secondments Phase 2: After Action Review group discussions with KT teams (including members of all groups covered earlier in interviews, *n* = 6) to discuss progress of KT projects; semi-structured interviews with three healthcare managers at end of KT projects explored the impact of secondments on the organisation, staff and patients	The six themes that emerged were KT skills development, effective workload management, team working, achieving KT objectives, enhanced care delivery, and enhanced education delivery Academic secondees reported increased research skills around KT theory and evaluation, and this was cited as an important aspect of the course; furthermore, participants enjoyed having developed these skills in a supportive team environment with mentoring from experienced KT team members	Secondments may be a useful way of increasing KT capacity for individuals and organisations The approach to KT capacity-building used in this study highlights the potential for experiential learning, the importance of mentorship and fostering a supportive training environment for participants to learn and develop new skills, and the need for flexibility to manage the duties of their concurrent roles	IV
Jones et al. (2015) [31]	Course attendees (number not stated)	Before and after evaluation (self-report) (not described in the report)	No data presented Authors reported that all course components were rated highly for quality relevance and usefulness; increases in participants’ self-reported understanding and confidence in KT theory, planning and communications were noted	The course appears to be relevant and useful and may be able to build researcher skills and confidence in KT and exchange	Report/Expert opinion; results from the pre–post evaluation were not reported
Santacroce et al. (2018) [32]	University of North Carolina pre- and post-doctoral research nurses (‘nurse scientists’) Number not stated	None reported The report describes a plan for KT competencies to be integrated into a research training program Students will be graded as part of their PhD assessment on research translation skills, in addition to other assessments, including dissertation completion and defence Pre- and postdoctoral trainees will be required to demonstrate competence in two of the six key themes of research translation taught as part of their training during their presentations, publications, research proposals and completed research	None reported	This style of KT training for researchers, embedded in a pre- and postdoctoral nurse scientist program, offers a potential model for others to follow; it demonstrates progress towards institutions recognising that KT work should be part of everyday research practice	Report of planned KT activities
Uneke et al. (2012) [33]	87 participants, including health researchers, health program managers, heads of departments in the health ministry and managers of health-based NGOs	Pre–post evaluation and a focus group were conducted with all participants; additionally, a survey was conducted with the senior researchers who participated in the workshop (*n* = 6) Pre–post self-report questionnaire rating knowledge of health policy-making processes, own capacity to use evidence and knowledge of evidence-informed policy-making The focus group focused on participants thoughts regarding ‘bridging the gap between health policy-makers and researchers’	Pre–post survey data was reported as means, medians and ranges Increases in self-reported knowledge and understanding of the health policy-making process were observed in all questionnaire items when compared to participant pre-workshop scores; items that showed the greatest change between the pre- and post-workshop condition were items related to knowledge of terms such as ‘policy brief’ or ‘types of evidence used for policy-making’ Thematic analysis of focus group data indicated a need for researchers to be more aligned to health systems and policy challenges, and to consider policy-maker perspectives in their work; furthermore, participants suggested increased collaboration between researchers and policy-makers could facilitate researchers informing policy-makers of relevant research as it becomes available; suggested models of partnership were either involving policy-makers in the planning of the research or including researchers in the implementation of policy programs	The findings suggest that a 1-day workshop training event for policy-makers and researchers may improve knowledge and understanding of key topics related to partnership research, evidence-informed policy-making and may enhance policy-makers’ research capacity; furthermore, the success of the workshop suggests that facilitating platforms to allow researchers and policy-makers to come together may be an avenue to help bridge professional divides and create the basis for future professional collaboration	IV
Uneke et al. (2018) [34]	10 researchers and 10 senior policy staff	Quantitative cross-sectional survey, a pre–post workshop survey and qualitative interviews Cross-sectional survey questions focused on themes around knowledge of secondments, and the role secondments can play in building capacity for organisations and individuals in evidence-based policy and building partnerships for ongoing collaboration Answers were recorded via a 5-point Likert scale where 1 = grossly inadequate and 5 = very adequate; values were reported as mean rating, median rating and range 6 participants (3 policy-makers and 3 researchers) were interviewed about their experiences and commitment to evidence-informed policy-making in the Nigerian context The pre–post workshop survey assessed self-reported knowledge of ‘the meaning of policy’, ‘knowledge of policy analysis’ and ‘knowledge of policy review process’	Both policy-makers and researchers strongly agreed that secondments offer the opportunity to enhance personal development and working practices and should be implemented on a continuous basis; they further felt that secondments enhanced capacity development, understanding of context and effective problem solving The establishment of a ‘Society for Health Policy Research and Knowledge Translation’ following the secondments provides evidence of the success of the secondments in fostering professional relationships; this society will function as a structure to promote ongoing evidence-based policy work in Nigeria Qualitative interviews with researchers indicated that the program made clearer the need to partner with policy-makers more to enhance evidence-based policy work and collaboration	Two-way approach acknowledges the collaborative and multidisciplinary nature of KT work Provides evidence of secondments between research and policy organisations being acceptable to participants, and useful as a training and organisational capacity-building exercise	IV
Uneke et al. (2018) [35]	*n* = 45 Researchers from the Implementation Research Team, policy-makers (from the Ministry of Health, Local Government Area, state primary healthcare development agency) and representatives from non-governmental organisations	Pre–post questionnaire design Participants were questioned pre- and post-workshop for 47 questions relating to understanding of content (using a 4-point Likert scale); pre–post scores were reported as group means, and change was reported by percentage mean increase There were also 3 questions about the workshop generally that were taken as single measures at the end of the final day; these questions tested acceptability of the facilitator, course content, and participant perceptions of the duration of the program	All 47 domains in the pre–post testing increased after the training workshop; values varied per topic The mean understanding of content range was 2.04–2.94 pre-workshop and 3.10–3.70 post-workshop; the lowest percentage mean increase in group understanding was 13.3 for ‘knowledge about managing political interference in policy-making and implementation’ and the greatest percentage mean increase in group understanding was 55.2 for ‘Understanding of iKT and eKT’ As for general enjoyment and acceptability of the workshop, the three final question mean results (on a 4-point Likert scale) were 3.79, 3.55 and 2.93	The program was effective in providing an acceptable program of KT learning aimed at researchers and policy-makers working in Maternal and Child health in Nigeria; there were strong self-reported increases in understanding across a broad range of KT areas after the course; the 3-day training workshop brought policy-makers and researchers together, which may enhance partnership working in the future	IV
Kho et al. (2009) [36]	5 participants in the 2008 KTSI (out of a total of 30)	Participant reflections	The mix of different learning formats was appreciated, the small group learning activity was viewed as particularly valuable, participants were able to build important relationships with other participants and faculty (all leaders in KT) and intended to maintain them, faculty enthusiasm was considered key to success, and participants appreciated the mentorship and career advice offered Suggestions for improvement were more time for informal networking and discussion, more emphasis on qualitative methods and health economics in KT and how KT can be applied in other aspects of health, e.g. educational Lessons learned were that KT is interdisciplinary and collaborative, negotiation skills are integral, the KT process is complex, confusing and multifaceted, and that it is crucial to use the most rigorous methods of inquiry	The KTSI was considered successful and beneficial by participants; it appeared to be successful to include participants from a range of disciplines and maintain the focus on adult learning and active learning; more interaction with faculty and career advice were considered desirable as was a greater emphasis on exploring the complementarity of qualitative and quantitative measures and more assistance in facilitating ongoing communication between participants and faculty The KTSI provided a networking opportunity for participants with shared interests in KT research and practice and gave them the chance to share ideas and resources	IV
Leung et al. (2010) [37]	One group of trainees from the KTSI authored the publication; number not stated	Trainee (author) reflections on, and description of, a training exercise	Outcomes were reported as participant experiences of the session; participants described the process of mapping the goals, target audience and message for the KT strategy as being useful in assisting their skill development	Through the process of planning out an end-of-grant KT strategy, participants were exposed to the challenges of developing KT initiatives, including lack of information about the specific project; they conclude the report by recommending that, as is increasingly sought after by research funders internationally, eKT strategies (and appropriate allowances in the budget) should be considered at the very beginning of research project planning, instead of as an afterthought at the end	Report/expert opinion
Bhogal et al. (2011) [38]	One group of trainees from the KTSI authored the publication; number not stated	Trainee (author) reflections on, and description of, a training exercise	Participants identified several key themes from their learning experience: ‘Balancing engaging stakeholders with moving forward’, ‘Exploring the role of the knowledge-to-action framework’, ‘Identifying KT research gaps’ and ‘Investigating methodological approaches for KT interventions and research’	Small group practice-based learning activities can expose participants to the challenges of KT practice in a controlled environment where they can learn in collaboration with peers; such activities may be a useful complement to traditional seminars covering theoretical background knowledge	Report/expert opinion

*CLAHRCs* Collaborations for Leadership in Applied Health Research and Care; *eKT* end-of-grant knowledge translation; *IKT* integrated knowledge translation; *KT* knowledge translation; *KTSI* Knowledge Translation Summer Institute; *NHMRC* National Health and Medical Research Council

Gerrish and Piercy [30] reported a qualitative evaluation of their secondment KT capacity-building program in the United Kingdom, involving focus groups and semi-structured interviews with secondees, KT project managers, KT teams and healthcare managers to assess the success of the program from a variety of perspectives. Pre–post surveys were used in four of the programs [31, 33–35] to assess confidence in KT knowledge and skills gained. For the two-way secondment program described by Uneke et al. [34], the pre–post survey was implemented during a policy dialogue event held at the completion of the secondments; qualitative and cross-sectional data were collected to assess participant experiences of the secondment.

The papers included in this review present preliminary indications of acceptability and appropriateness of the capacity-building initiatives described but do not provide rigorous evidence to support their effectiveness. The overall quality of evidence included in this review is rated as ‘very low quality’ according to GRADE criteria [39], meaning that the evidence is very uncertain. Five of the papers under the NHMRC rating scale would individually be ranked as ‘level IV’ evidence (the lowest level included in this rating scale, where systematic reviews of randomised controlled trials are given a level ‘I’ rating). This is due to the low-quality quantitative data reported in these five studies. The remaining four papers are considered qualitative reflections or reports and thus are not assessable by the NHMRC scale.

### Research outcomes

All of the training initiatives included in this review were described as beneficial, although, as noted above, the level of evidence was low. From the qualitative evaluation reported in Gerrish and Piercy [30], participants, hosting teams and KT managers described the secondments as a success due to the reportedly improved performance of trainees in KT facilitation and evaluation skills. This improvement was believed to be due to the practical skills they developed while embedded in the KT team as well as the mentorship they received from senior team members. In the secondment paper by Uneke et al. [34], researchers (qualitatively) reported having a deeper understanding of the need for researchers and policy-makers to work together and that the secondments offered the opportunity for personal and professional development. Jones et al. [31] and Uneke et al. [33, 35] found that participant self-reported knowledge of key outcomes increased post-workshop. Santacroce et al. [32] did not report outcomes in their report.

Participant reports emerging from the Knowledge Translation Summer Institute [36] suggest that the authors enjoyed the different learning formats used at the Institute, the brief mentoring sessions they had with faculty members and the chance to network with other early career researchers. The reports by Leung et al. [37], Bhogal et al. [38] and Kho et al. [36] all contribute to demonstrating the importance of a supportive learning environment when participants are first learning about KT.

## Discussion

The current study is, to the best of our knowledge, the first systematic review of published reports on capacity-building initiatives for KT and/or partnership research in the field of health research. While internationally there is growing interest in this area, we found the published literature on capacity-building programs for health researchers to be sparse, with only nine papers identified covering seven separate training initiatives. A variety of approaches were used for training in each of the programs; this heterogeneity combined with the minimal level of evaluation undertaken to date means it is not yet clear which training strategies in this area are likely to be the most effective.

The identified training initiatives all utilised a combination of different delivery styles to expose trainees to the multidisciplinary nature of KT, an approach that has previously been recommended in the literature [24].

### Training in practical KT skills

All the included training programs covered some of the practical skills needed for KT, including skills in KT planning and evaluation, relationship-building, and communication and teamwork; these skills have all been identified in the literature as important for KT or partnership research [19, 21–23]. All programs were face-to-face allowing participants the opportunity to network with other KT trainees or team members. Another key feature of many of the programs was opportunities to engage in practice-based learning through secondments [30, 34], working alongside mentors to assist with KT activities [32], or classroom-based practical activities [37, 38]. Practical learning exercises as well as instruction on relevant concepts and theory have been identified as important components of KT training by researchers in Canada, as well as more broadly in the adult-education literature [40, 41]. Brief mentoring sessions between participants and course faculty during the Knowledge Translation Summer Institutes appear to have been well received by participants [36], and may be an additional method of training researchers in KT that merits further inquiry [42, 43].

Skills-based competencies related to collaboration and partnership, particularly with policy-makers, were also identified in the programs described by Jones et al. [31] and Uneke et al. [33, 34]. This type of training aligns with skills described by Mallidou et al. [23] such as ‘sharing knowledge’, ‘collaboration and teamwork’ and ‘knowledge brokering’. This may be an important theme for building researcher capacity for KT, as research suggests that these relationship-building skills are important in creating policy-focused research and in contributing to greater research impact [13, 44, 45].

### Training in KT theory

All of the training initiatives that utilised a traditional course structure (workshop over one or more days) included content on the theoretical background of KT [31, 33, 35, 36]. Such training has been highlighted as critical in Mallidou et al.’s [23] KT key competencies framework, in particular, understanding of context, research processes, evidence resources and dissemination. In addition to teaching researchers about the theory of KT, many of the programs included in this review also provided information about health policy-making processes to increase participants’ knowledge of the contextual aspects of KT work. The inclusion of both theoretical aspects of KT and practical skills-based aspects of KT in all training programs indicates a shared view that participants need to be exposed to a broad range of competencies to be able to effectively engage in KT.

### Evaluation of the KT programs and courses

As training initiatives in KT or partnership research are still in their infancy, it is unsurprising that the publications we found reported exploratory evaluations of, or reflections on, single rounds of training initiatives rather than large scale evaluations. Participant experience, self-report surveys and qualitative evaluation methods were used to describe the successes and failures of most programs.

Broadly speaking, all studies reported that their approach to capacity-building in KT was well received and (where measured) that the program improved participant confidence in key skills related to KT. While there is not yet strong evidence on which to base decisions about which training strategies might be most effective in this field, the papers we located have provided useful insights into the types of approaches interested parties might take to developing skills-building initiatives in this area.

As with any review, we may not have captured every relevant paper, especially due to the diversity of terms used in the field of KT [46]. In addition to these limitations, the authors acknowledge that the training initiatives identified in published articles are likely to represent a very small sample of the body of work that is being carried out in this field. Nevertheless, to the best of our knowledge, this review represents the first systematic review of available peer-reviewed literature on the subject of KT/partnership research capacity-building programs for researchers.

## Conclusion

The reports found in this review provide early indications of potentially relevant learning objectives, themes and approaches in increasing researchers’ capacity to engage in KT work. Promising training themes include increasing researchers’ knowledge and understanding of health policy-making processes, improving understanding of KT research methods and KT theory, improving communication and relationship-building skills, and skills around the design and evaluation of KT plans. As this area of research matures, it is hoped that more in-depth evaluations of capacity-building programs for KT and partnership research are conducted, allowing the establishment of a stronger evidence base to guide program development.

## Data Availability

Please contact author for data requests.

## Abbreviations

IKT: integrated knowledge translation
KT: knowledge translation
NHMRC: National Health and Medical Research Council
